# A meta-analysis of renal outcomes in living kidney donors

**DOI:** 10.1097/MD.0000000000003847

**Published:** 2016-06-17

**Authors:** Sha-Sha Li, Yan-Mei Huang, Min Wang, Jian Shen, Bing-Jie Lin, Yi Sui, Hai-Lu Zhao

**Affiliations:** a Center for Diabetic Systems Medicine, Guangxi Key Laboratory of Excellence; b College of Clinical Medicine, Guilin Medical University, Guilin, China.

**Keywords:** albuminuria, end-stage renal disease, glomerular filtration rate, kidney transplantation, living kidney donor

## Abstract

Given the increased burden of end-stage renal disease (ESRD), renal outcomes of kidney donation by living donors are of particular interest. PubMed, ProQuest, MEDLINE, EMBASE, Chinese national knowledge infrastructure, and Wanfang databases were searched for clinical outcomes of living kidney donors (LKDs) including renal death, ESRD, proteinuria/albuminuria, and renal function after donation. We included 62 studies from 19 countries involving 114,783 kidney donors and nondonors to evaluate the renal consequences less than 6 months, 6 months to 5 years, 5 to 10 years, and 10 years onward after donation. The pooled data showed that uninephrectomy significantly decreased glomerular filtration rate and creatinine clearance rate in parallel with increased serum creatinine concentration (all *P* < 0.05). The drastic changes in renal function occurred within 6 months rather than 5 to 10 years after donation. Ten years and onward, rate of proteinuria/albuminuria increased gradually: microalbuminuria from 5.3% to 20.9%, proteinuria from 4.7% to 18.9%, and overt proteinuria from 2.4% to 5.7% (all *P* < 0.05). Prevalence of ESRD was 1.1%. All-cause mortality was 3.8% and all the renal deaths on average occurred 10 years postnephrectomy. LKDs might have aggravated glomerular filtration and creatinine clearance within 6 months after donation. Five years and onward, albuminuria, proteinuria, ESRD, and death might be the major concerns of LKDs. Long-term studies may clarify the survival time after donation.

## Abstract 2

Supplemental Digital Content is available in the text

## 1 Introduction

Patients with end-stage renal disease (ESRD) outnumber deceased kidney donors available for transplantation.^[1]^ Living donor kidney transplantation becomes an important option for ESRD treatment, owing to prolonged waiting times on transplant list, superior outcomes for recipients, and evolving criteria for donor acceptance.^[2–4]^ Increasing transplantations should not mean increasing risk to donors. A recent study highlights an increased cumulative incidence and lifetime risk of ESRD following donation.^[5]^ Previously, we have reported that uninephrectomized rats progressively developed renal impairments and glomerulosclerosis accompanied by insulin resistance, hyperglycemia, hyperlipidemia, fat redistribution, and remnant kidney cancer.^[6–11]^ Definitive outcome assessment is precluded by lacking of a comprehensive system or registry for follow-up. Safety remains in obscurity because of the inferences at single centers with limited generalizability, restrictive sample size, and inappropriate comparison groups.^[12]^ All these findings generate concerns about postnephrectomy outcomes with special focus on the remnant kidney. Therefore, we conducted a systematic review and meta-analysis to investigate the short-, mid-, and long-term changes in renal function relative to proteinuria/albuminuria, ESRD, and mortality in living kidney donors (LKDs).

## 2 Methods

The Preferred Reporting Items for Systematic Reviews and Meta-Analyses Statement was used as a guide in the present study that ensures a standard method for transparent and complete reporting of systematic reviews and meta-analyses.^[13]^ The present study was approved by the Ethics Committee Board of Guilin Medical University (GLMC20120308HL). We had reviewed each included studies and found 19 studies mentioned in the methods section that ethical approval and written informed consent were obtained.

### 2.1 Search strategy

Four reviewers (LSS, HYM, SJ, and LBJ) systematically searched 5 English databases including PubMed, ProQuest, Cochrane Library, MEDLINE, and EMBASE; 4 Chinese and Japanese databases including Wanfang database, Chinese National Knowledge Infrastructure, Chinese Biomedical Literature Database, and Japan Science and Technology Information Aggregator Electronic; and other electronic databases including the United Network for Organ Sharing and Organ Procurement and Transplantation Network. The search terms “living kidney donation,” “living kidney transplantation,” “renal transplantation,” “nephrectomy,” and “unilateral nephrectomy” were used in various combinations with “renal outcomes,” “renal function,” “kidney function,” “creatinine clearance rate,” “serum creatinine,” “plasma creatinine,” “glomerular filtration rate,” “proteinuria,” “albuminuria,” “ESRD,” “mortality,” and “death.” In addition, relevant studies were also identified through manual search of the bibliographies and reference lists.

### 2.2 Eligibility criteria

All published articles had to meet the following inclusion criteria^[1]^: original interventions were conducted with comparing renal outcomes before and after donation or between donors and nondonors^[2]^; available data were the remnant kidney outcomes including glomerular filtration rate (GFR), estimated GFR, creatinine clearance rate (Ccr), serum creatinine (sCr), and urinary protein excretion^[3]^; reports showed rate of mortality, ESRD and proteinuria/albuminuria, or reports disclosed sufficient data to calculate these values; and^[4]^ one of 4 postnephrectomy durations was defined by <6 months (short term), 6 months to 5 years (mid-term), 5 to 10 years (prolonged term), and >10 years (long-term). For the LKDs, time at risk was accrued from the date of uninephrectomy. Nondonors were accrued from the enrollment into study. All potential articles were in English or Chinese and published in their entirety. If there are multiple publications from the same 1 investigation, we cited the most representative publication with largest number of donors and longest time of follow-up.

Literatures meeting the following criteria were excluded: nonclinical nature, duplication, studies that did not investigate duration after donation as a variable or renal function as an outcome, nonhuman studies, unclear of outcome evaluation, and nonoriginal reports including reviews, editorials, letters, and commentaries. The chance-corrected agreement between 4 reviewers for study inclusion was applicative (kappa = 0.87).

**Figure 1:**
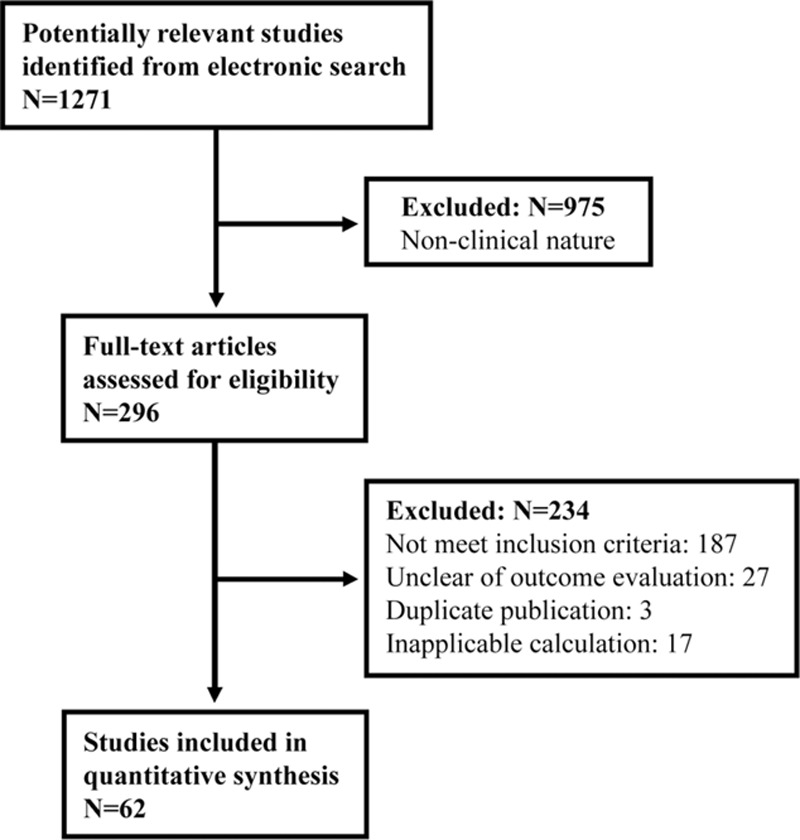
Flow chart of study selection process.

Initially, we downloaded 1271 full-text articles of potential studies, of which 975 publications were excluded due to nonclinical nature (Fig. 1). After detailed evaluation, 234 more were subsequently excluded according to our inclusion and exclusion criteria. Eventually, 62 studies published from 1973 to 2014 and from 19 countries involving a total of 114,783 participants were included in this meta-analysis.

### 2.3 Data extraction

Four coauthor of this study (LSS, HYM, SY, and SJ) independently extracted the data from the 62 eligible studies. The extracted data were as follows: study descriptions, participants’ characteristics, follow-up duration after donation, renal function measurements, and methods of these measurements and calculation (Tables 1 and 2). To avoid age-related kidney dysfunction after donation, we conducted comparison of long-term outcomes between donors and nondonors.

**Table 1:**
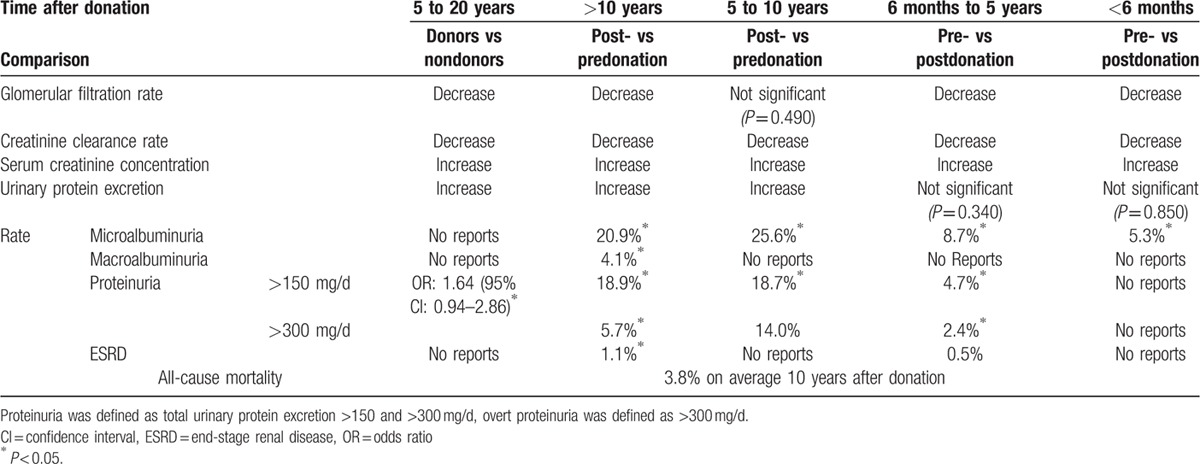
Summary of renal outcomes after donation.

**Table 2:**
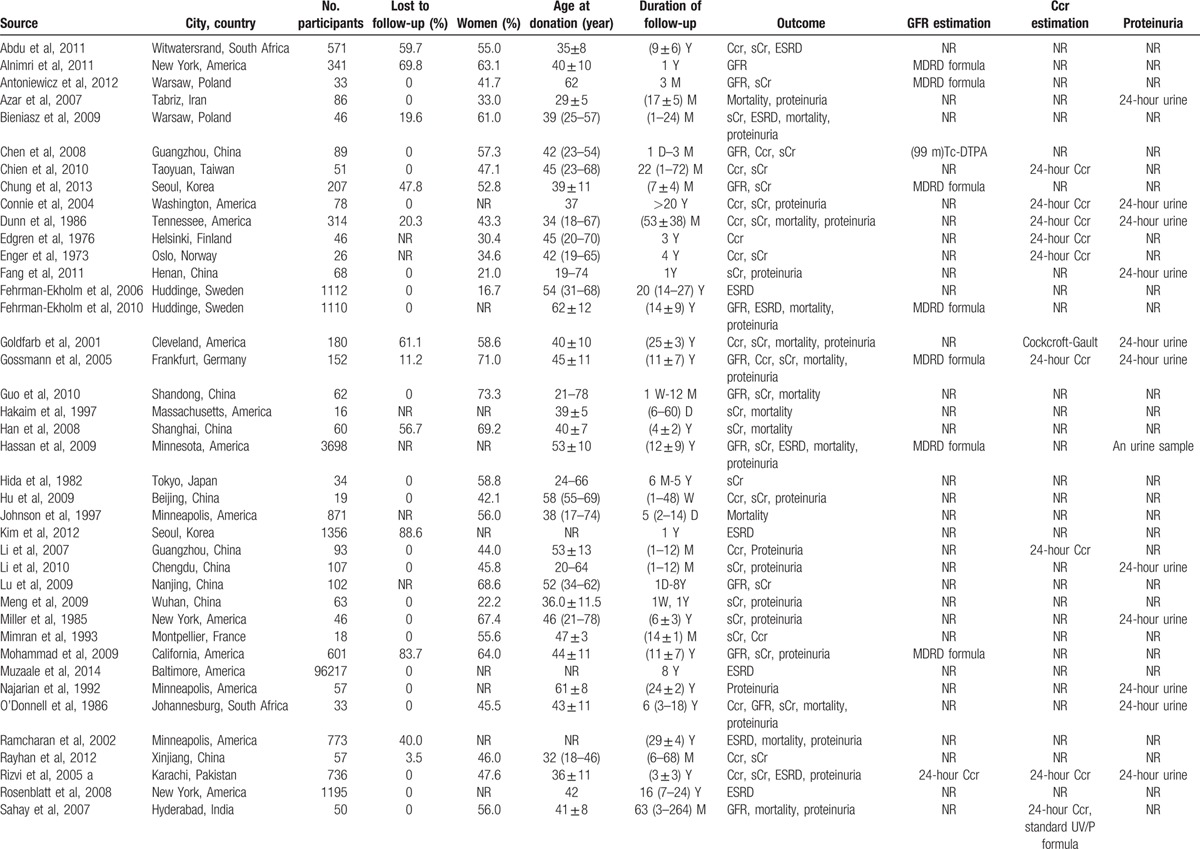
Characteristics of the 63 studies included in the meta-analysis.

**Table 2 (Continued):**
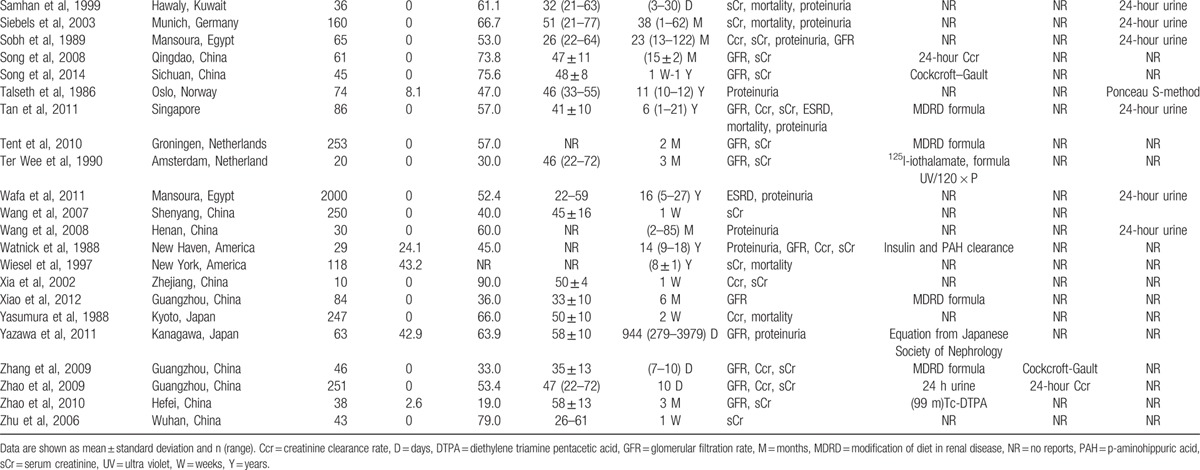
Characteristics of the 63 studies included in the meta-analysis.

### 2.4 Outcome measures

The primary outcomes included rates of mortality, ESRD, and proteinuria/albuminuria. The secondary outcomes were the remnant kidney function parameters such as GFR, Ccr, and sCr.

### 2.5 Validity assessment

We used the risk of bias assessment tool for nonrandomized studies to evaluate the quality of the included studies for the purpose of reliability, feasibility, and validity.^[14]^ The risk of bias assessment tool for nonrandomized studies tool tests the selection of participants, confounding variables, measurements of intervention, blinding of outcome assessments, incomplete outcome data, and selective reporting.

### 2.6 Statistical analysis

The fixed-effect or random-effects models with generalized least-squares estimation were used to calculate the summarized mean estimates. *Q*-test was used to compare the mean effect between different duration after donation. In order to explore the potential sources of heterogeneity, subgroup meta-analyses and meta-regression analyses were conducted based on participants’ gender, age, geographic region, measurements, and quality of the studies. Additionally, we also conducted sensitivity analyses to assess the robustness in this study.

The heterogeneity among the literatures was examined using *I*^2^ statistics. *I*^2^ < 50% indicates low heterogeneity and fixed-effect model as appropriate, random-effects model on the contrary. Publication bias was assessed by visual inspection of funnel plot and then tested by the Egger regression and trim and fill analyses. The *P* values for the Egger test are less than 0.05 in the presence of publication bias. All of the statistical analyses were performed using the Review Manager 5 software package (version 5.1; The Nordic Cochrane Center, Copenhagen, Denmark) and Stata 11.0SE statistical software package (StataCorp, College Station, TX).

## 3 Results

### 3.1 Study description and quality and bias assessment

#### 3.1.1 Study characteristics

Tables 1 and 2 show the 62 studies included 114,783 participants. Among the 62 studies (Table 2 ), 62 showed comparison between predonation and postdonation (GFR in 23, Ccr in 22, sCr in 43, and urinary protein excretion in 6), while 8 had comparison of donors and nondonors (GFR in 4, Ccr in 5, sCr in 6, and urinary protein excretion in 3). Rate of ESRD, albuminuria/proteinuria, and mortality were documented in 12, 26, and 19 studies, respectively.

In general, 72.6% commendably followed the total number of donors, 47.3% depicted the characteristics of donors lost to follow-up, 38.4% described types of surgery, and 74.7% had scheduled renal outcomes measured. Definitions of albuminuria/proteinuria were reported in 77.2%, and criteria for ESRD were described in 52.3%. Details of measuring GFR, Ccr, sCr, and urinary protein excretion were found in 87.0%, 54.5%, 97.3%, and 87.4%, respectively.

### 3.2 Methodological quality and bias of studies

**Figure 2:**
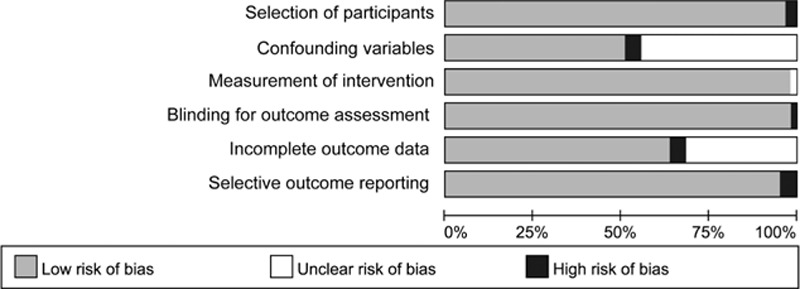
Risk of bias graph of all included quasi-randomized controlled trials using the risk of bias assessment for nonrandomized studies (RoBANS) tool.

In this meta-analysis of the 62 studies, the risk of bias analysis revealed concerns about low- versus high-risk of bias for selection of participants (96.8% vs 3.2%), confounding variables (57.1% vs 4.8%), measurements of intervention (98.4% vs 0), blinding of outcome assessments (98.4% vs 1.6%), incomplete outcome data (63.5% vs 4.8%), and selective reporting (95.2% vs 4.8%), as shown in Fig. 2 and Supplemental Table 1.

The vast majorities of the funnel plots assessed by Egger regression test and trim and fill analysis showed no significant publication bias (Table 3).

**Table 3:**
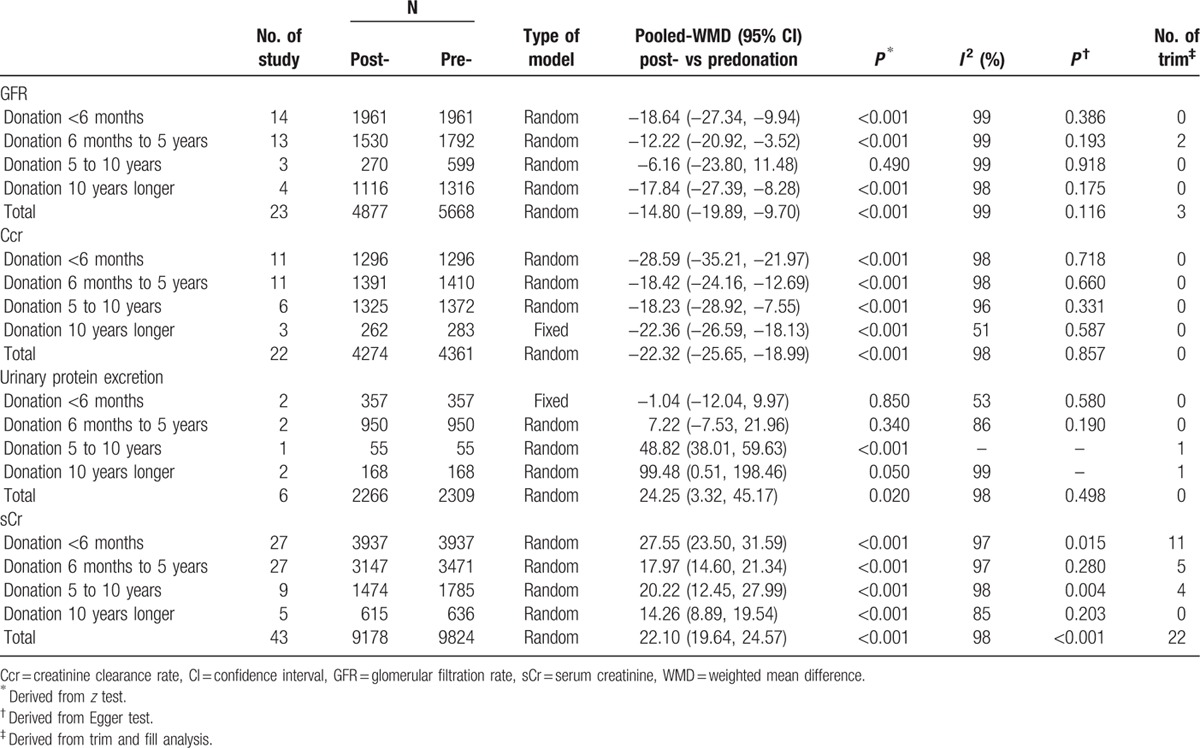
Changes in donors’ renal function in relation to duration after donation.

### 3.3 Changes of renal functions between pre- and postdonation

Table 3 summarizes the outcomes along with time after donation. A random-effect model was selected due to the heterogeneity of reporting GFR (*I*^2^ = 99%), Ccr (*I*^2^ = 98%), urinary protein excretion (*I*^2^ = 98%), and sCr (*I*^2^ = 98%). Pooled analysis revealed a significant reduction of GFR (weighted mean difference [WMD], −14.80; 95% confidence interval [CI], −19.89 to −9.70) and Ccr (WMD, −22.32; 95% CI, −25.65 to −18.99) in parallel to elevation of urinary protein excretion (WMD, 24.25; 95% CI, 3.32–45.17) and sCr (WMD, 22.10; 95% CI, 19.64–24.57).

**Figure 3:**
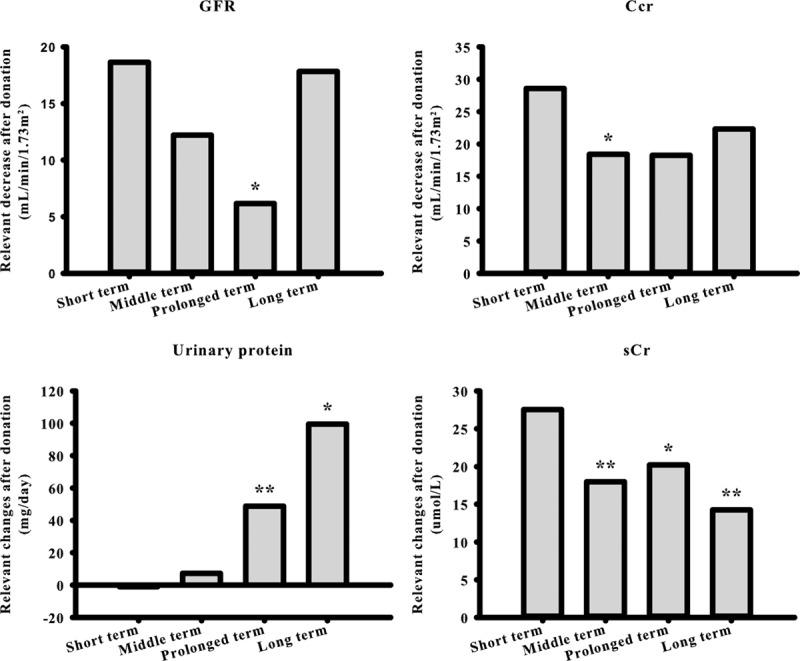
Changes of renal function in relation to different duration after donation. *P* value derived from *Q*-test by comparing with short-term group. ^∗^*P* < 0.05, ^∗∗^*P* < 0.001. Ccr = creatinine clearance rate, GFR = glomerular filtration rate, sCr = serum creatinine.

Consistently, the largest absolute number of WMD for GFR, Ccr, and sCr generated within 6 months postnephrectomy while urinary protein excretion progressively aggravated along with time after donation (Table 3, Fig. 3).

### 3.4 Comparison of renal functions between donors and nondonors

Eight studies included 792 donors and 562 nondonors 5 to 20 (mean 10) years after donation. Table 4 shows the donors contrasting nondonors to have decreased GFR and Ccr in parallel to increased sCr and urinary protein excretion (all *P* < 0.031). Funnel plot was detected by Egger test and trim and fill analysis (Table 4).

**Table 4:**
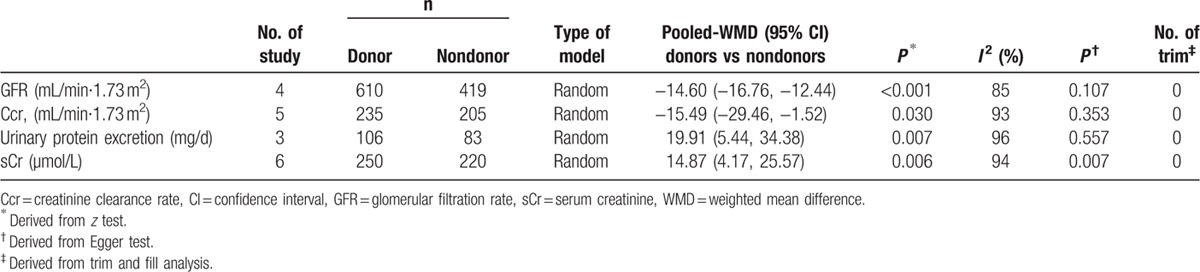
Changes in renal function between donors and nondonors 5 to 20 years after donation.

### 3.5 Rate of proteinuria postdonation

The cut-off points and rates of proteinuria and albuminuria in relation to donation were given in 26 studies of 5337 LKDs. Table 1 shows that rate of microalbuminuria, proteinuria, and overt proteinuria increased along with time after donation (*P* < 0.050).

### 3.6 Rate of ESRD postdonation

Rate of ESRD was described in 12 studies. A total of 516 donors had defined ESRD diagnosed 14 ± 9 years after donation. In general, total pooled rate of ESRD was 1.1% 10 years onward and 0.5% 6 months to 5 years after donation (Table 1).

### 3.7 Mortality after donation

Nineteen studies of 8098 donors addressed total mortality after donation. All-cause mortality was reported less than 10.0% in the majority of studies. The pooled overall mortality was 3.8% (95% CI, 1.15%–6.45%). Nephrectomy-related deaths were extracted from 15 studies involving 5301 donors. Among 19 studies reporting mortality, 2 studies revealed deaths attributable to renal failure. The pooled renal death rate was 0.3% and the renal deaths on average occurred 10 years after donation. One donor died of renal failure 32 years after nephrectomy at the age of 76.^[15]^

### 3.8 Potential sources of heterogeneity and sensitivity analyses

Subgroup analysis (Table 5) and meta-regression analyses (Table 6) disclosed sex, age at donation, and study location as potential sources of between-study variance in this study. Age at donation could account for 24.4% of the heterogeneity for Ccr and 18.6% of the heterogeneity for sCr. Moreover, 61.2% of the heterogeneity for urinary protein excretion could be explained by study location. In contrast, sex, age at donation, and study location had no significant impact on the heterogeneity exploration of GFR (Table 6).

**Table 5:**
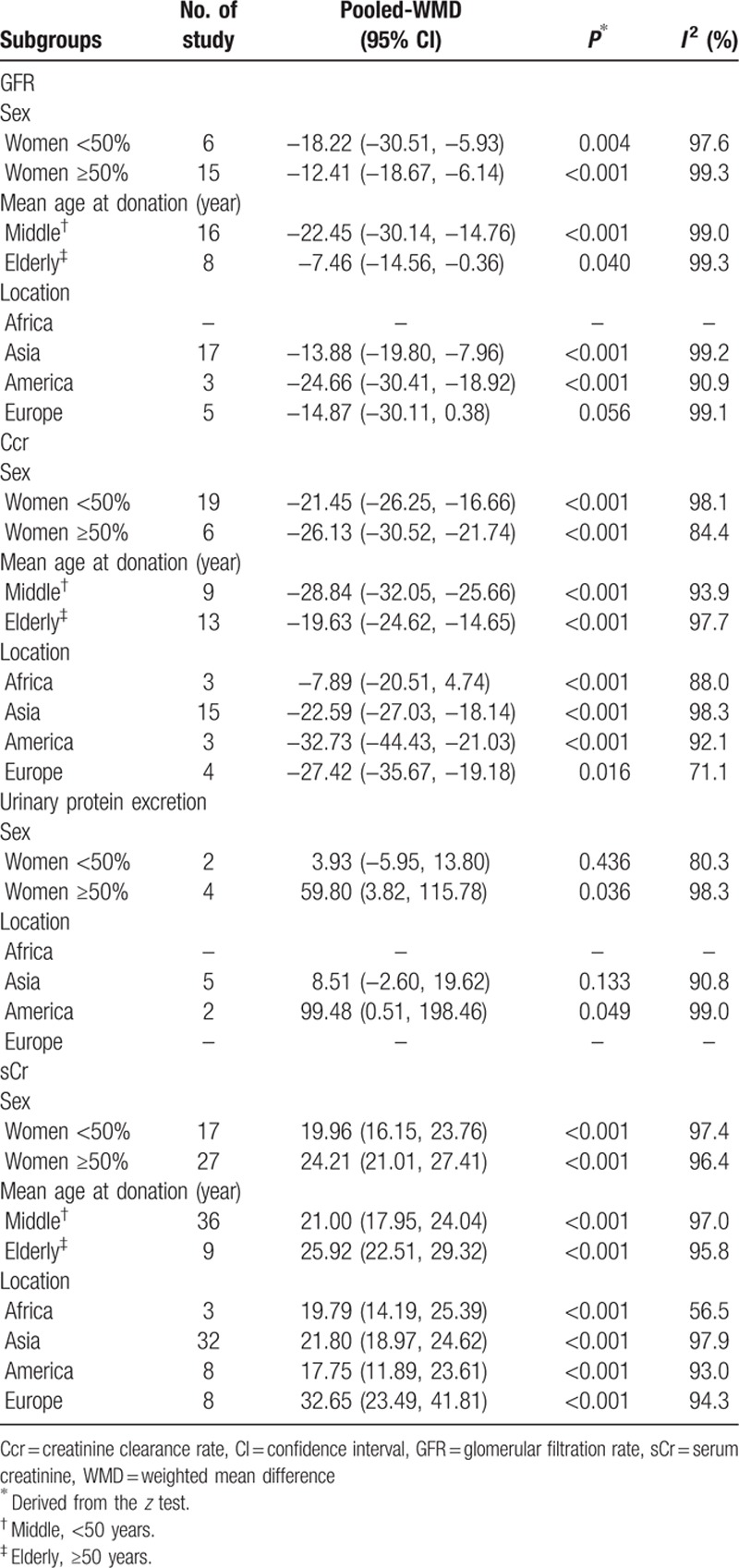
Subgroup analyses to explored sources of heterogeneity.

**Table 6:**
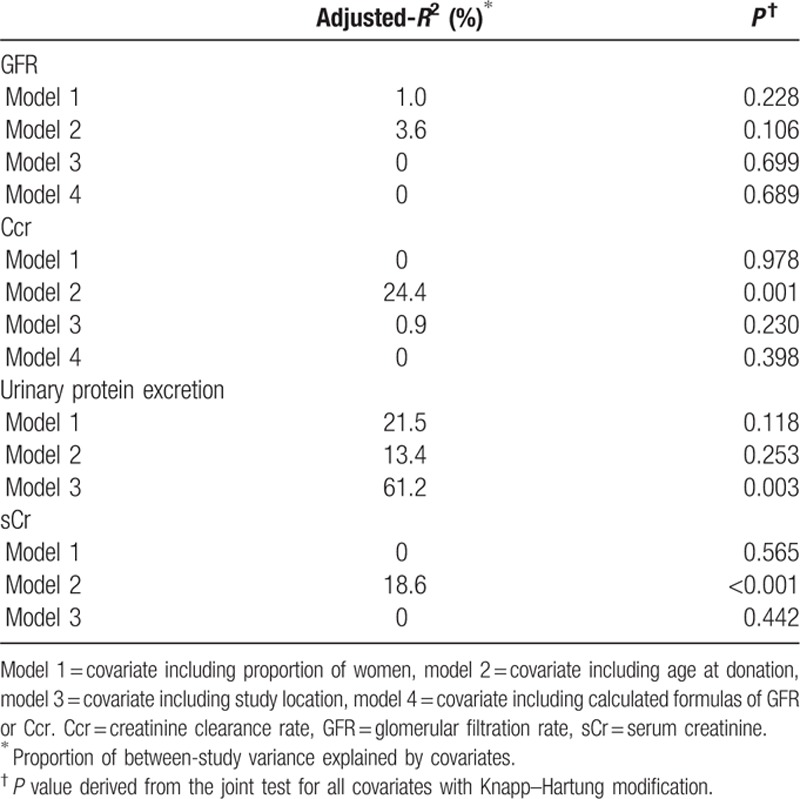
Meta-regression to explored sources of heterogeneity.

After exclusion of 3 studies that had a low risk of bias, sensitivity analysis yielded similar results of Ccr, GFR, sCr, and urinary protein excretion after donation (data not shown). Stepwise elimination of the studies was also used in the sensitivity meta-analysis. Overall, the sensitivity analysis yielded a nearly identical set of pooled WMD for Ccr, GFR, sCr, and urinary protein excretion (Fig. 4 ).

**Figure 4:**
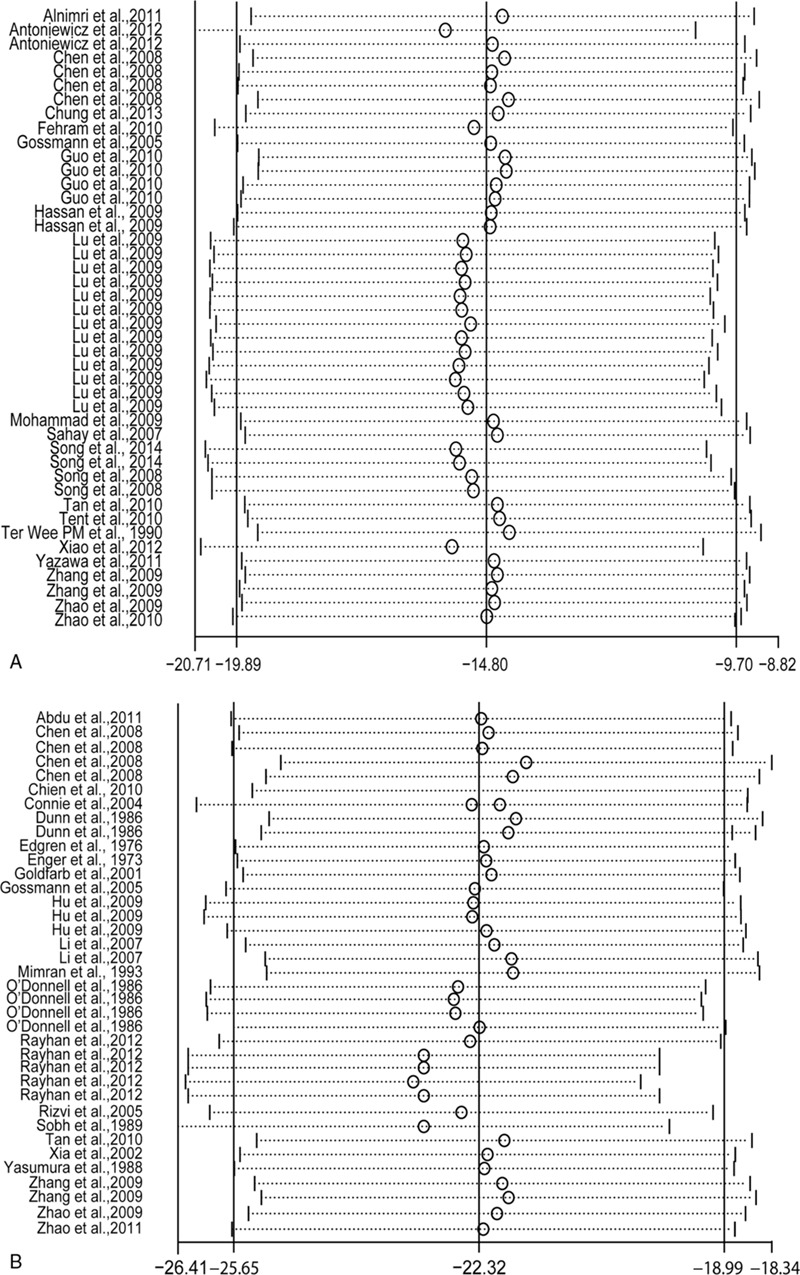
Sensitivity analyses for renal functions (A) glomerular filtration rate, (B) creatinine clearance rate, (**C)** urinary protein excretion, and (D) serum creatinine.

**Figure 4 (Continued):**
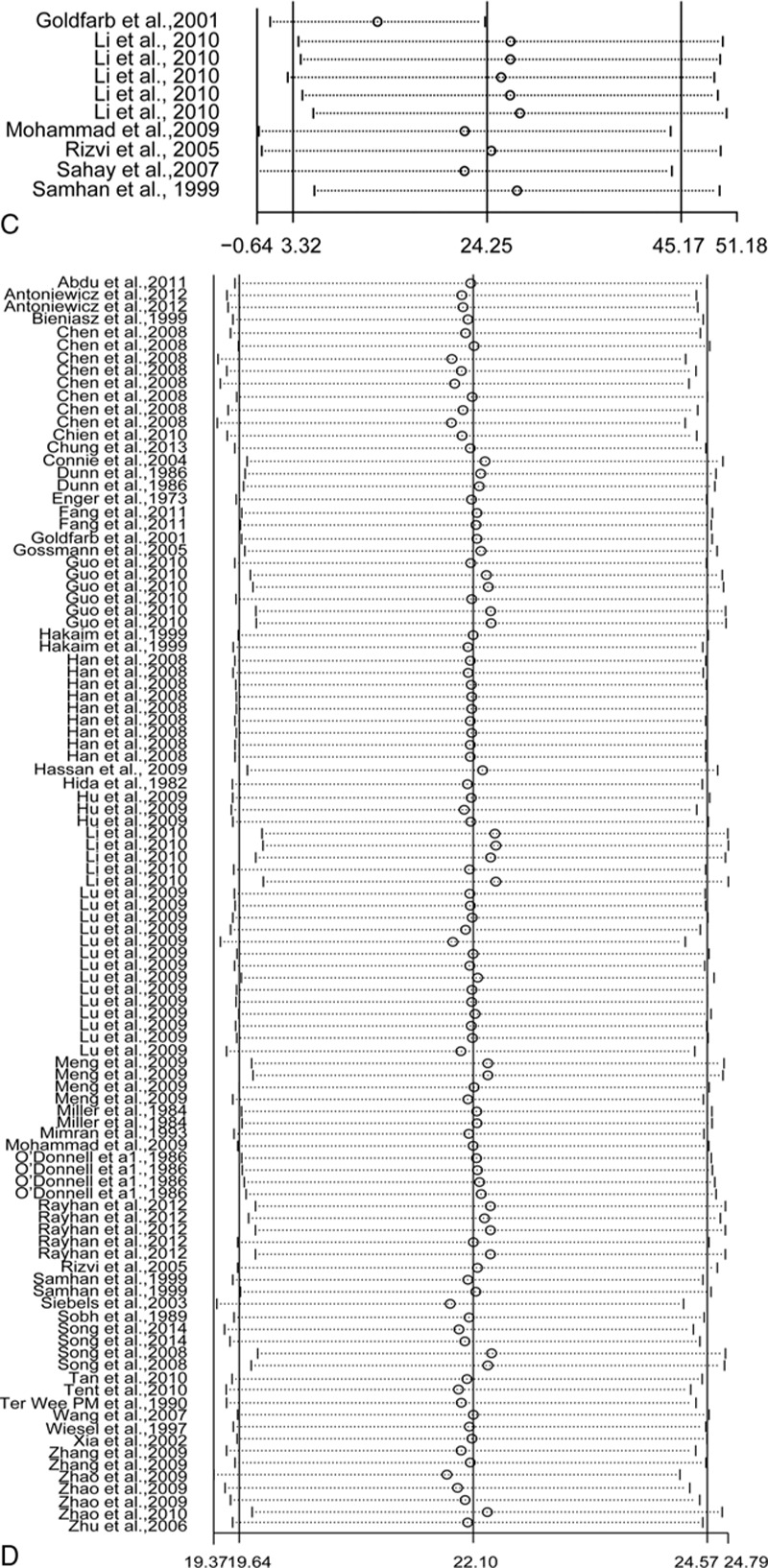
Sensitivity analyses for renal functions (A) glomerular filtration rate, (B) creatinine clearance rate, (**C)** urinary protein excretion, and (D) serum creatinine.

## 4 Discussion

### 4.1 Summary of findings

Findings from this study of LKDs are as follows^[1]^: donation-induced renal dysfunction is evident by decreased GFR and Ccr in parallel to increased urinary protein excretion and sCr concentration^[2]^; the drastic change in the donors’ renal function consistently occurs within 6 months after donation rather than 6 months to 10 years postnephrectomy^[3]^; the rate of microalbuminuria, proteinuria, and ESRD gradually increase at 5-year intervals postkidney donation; and^[4]^ the LKDs would see less than 5.0% of overall morality and less than 1.0% of renal deaths on average 10 years after donation. In the present study, we also have performed subgroup analysis and meta-regression to validate the contribution of women proportion, age at donation, and study location to heterogeneity among the studies and between-study variance. In general, publication bias, as examined by funnel plots and sensitivity analyses, is unlikely in most studies included for this meta-analysis.

### 4.2 Interpretation of findings

The drastic renal dysfunction observed within 6 months after donation indicates incomplete compensation of the remnant kidney. It may take a few months for the remnant kidney to compensate for glomerular filtration and creatinine clearance. Indeed, there is a humoral substance acting specifically on the kidney that promotes renal compensatory hyperplasia in uninephrectomized rats.^[16]^ The renal compensatory hyperplasia may ameliorate the stressful demand for creatinine clearance at the cost of glomerular hyperfiltration subsequently followed by increased urinary protein excretion.

Increased urinary protein excretion results in albuminuria and proteinuria. In the present study, the rate of albuminuria and proteinuria aggravated along with time after donation (Table 1). Proteinuria is a well-known marker of disease progression.^[17]^ In this meta-analysis, the estimated rate of ESRD in donors was approximately 1.0%, higher than 0.1% to 0.5% reported in previous studies.^[5,18,19]^ In fact, a recent study has shown similar findings of increased risk of ESRD in LKDs.^[5]^ Moreover, the estimated lifetime risk of ESRD is higher in black donors than in white donors. Furthermore, the hazard of ESRD should not be neglected when considering conditions including older age, diabetes, obesity, and hypertension are no longer classified as absolute contraindications for living kidney donation.^[20,21]^

The lifetime impact of albuminuria/proteinuria should never be underestimated. Albuminuria/proteinuria is an important marker for endothelial dysfunction predisposing to the development of ESRD,^[22,23]^ cardiovascular disease,^[24–26]^ and cerebrovascular accident.^[27]^ ESRD may lead to renal deaths whereas cardiovascular disease and cerebrovascular accident will escalate all-cause mortality. Although there is cautious optimism concerning perioperative mortality, survival, and the risk of ESRD in carefully screened kidney donors,^[1,28,29]^ the lifetime risk for LKDs should be clarified in relation to coexisting medical conditions, age, gender, and race.^[20,30]^

In general population, renal function declines with aging. In the present study, comparisons between donors and nondonors have suggested donation-induced renal dysfunction echoed by the results of the paired comparisons (Tables 3 and 4). Although age is deliberately an important factor for renal outcomes, LKDs should aware the potential risks of donation-associated renal hyperplasia and deficiency.

### 4.3 Limitations and future studies

This study has potential limitations that may confound the results.^[1]^ In addition to time after donation, factors such as comorbidities, genetic predisposition, ethnic, and racial disparities may also influence donor's renal outcomes. A recent study has demonstrated that persons with metabolic syndrome are at an increased risk for ESRD and death.^[31]^ And, we plan further studies in this area considering more confounding factors to confirm this hypothesis.^[2]^ Each transplantation center has established methods for the measurement of GFR, Ccr, sCr, and urinary protein excretion. Here we used model 4, stratified by different calculation of estimated GFR and Ccr, heterogeneity remains unchangeable. Details in methodological description in many studies included in this meta-analysis are unknown. Moreover, estimated GFR calculated from the Cockroft-Gault and Modification of Diet in Renal Disease formulas are verified only in Caucasian population. This is particularly relevant when comparing GFR between worldwide donors. Although there is modified GFR estimating equation for Chinese patients with chronic kidney disease,^[32]^ whether the equation is appropriate for Chinese kidney donors remains uncertain.^[3]^ Albuminuria and proteinuria were not defined according to a uniform urine collection. A 24-hour urine collection was used in most of the included studies while a spot urine collection used in the others. Therefore, the rate of albuminuria and proteinuria in donors and nondonors may differ due to different urine collections. Hereby, we selected the studies using 24-hour urine collection for pooled analysis and the results unaltered.

In our future works, we will compare GFR estimated by the equations and GFR measured by the (99m)Tc-diethylene triamine pentacetic acid plasma clearance method. The mechanisms underlying uninephrectomy-induced glomerular hyperfiltration and subsequent proteinuria are also of our interest.

## 5 Conclusions

LKDs may see renal deficiency aggravated within 6 months after donation, followed by an increased risk of proteinuria and ESRD 5 years and onward. These findings alert LKDs to avoid using renal toxic chemicals and to take cautious action for renal protection.

## Acknowledgements

The authors thank all the participants for their support in this study. The authors also thank Mr Golden Wilson for his English proofing.

## Supplementary Material

Supplemental Digital Content
