# Supplementary material for: A meta-analysis of renal outcomes in living kidney donors
Source: Medicine (Baltimore). 2016 Jun 17;95(24):e3847. doi: 10.1097/MD.0000000000003847 (PMC4998450; doi:10.1097/MD.0000000000003847)
Supplement: Supplemental Digital Content [file medi-95-e3847-s001.doc]

**Supplemental Tables**

**Table 1. Risk of bias assessment for non-standard studies**

| **Studies** | **Selection of participants** | **Confounding variables** | **Measurement of exposure** | **Blinding of outcome** | **Incomplete outcome data** | **Selective reporting** |
| --- | --- | --- | --- | --- | --- | --- |
| Abdu et al., 2011 | Low | Low | Low | Low | Unclear | Low |
| Alnimri et al., 2011 | Low | Low | Low | Low | Low | Low |
| Antoniewicz et al., 2012 | Low | Unclear | Low | Low | Unclear | Low |
| Azar et al., 2007 | Low | Unclear | Low | Low | Low | Low |
| Bieniasz et al., 2009 | Low | Unclear | Low | Low | Low | Low |
| Chen et al., 2008 | Low | Low | Low | Low | Low | Low |
| Chien et al., 2010 | Low | Unclear | Low | Low | Low | Low |
| Chung et al., 2013 | Low | Unclear | Low | Low | Low | Low |
| Connie et al., 2004 | Low | Unclear | Low | Low | Low | Low |
| Dunn et al., 1986 | Low | Low | Low | Low | Unclear | Low |
| Edgren et al., 1976 | Low | Unclear | Low | Low | Low | Low |
| Enger et al., 1973 | Low | Low | Low | Low | Unclear | Low |
| Fang et al., 2011 | Low | Low | Low | Low | Low | Low |
| Fehrman et al., 2006 | Low | Unclear | Low | Low | Low | Low |
| Fehrman et al., 2010 | Low | Low | Low | Low | Low | Low |
| Goldfarb et al., 2001 | Low | Low | Low | Low | Unclear | Low |
| Gong et al., 2011 | Low | Unclear | Low | Low | Low | Low |
| Gossmann et al., 2005 | Low | High | Low | Low | Unclear | High |
| Guo et al., 2010 | Low | Low | Low | Low | Low | Low |
| Hakaim et al., 1997 | Low | Unclear | Low | Low | Unclear | Low |
| Han et al., 2008 | Low | Unclear | Low | Low | Low | Low |
| Hassan et al., 2009 | Low | Low | Low | Low | Low | Low |
| Hida et al., 1982 | Low | Low | Low | Low | Unclear | Low |
| Hu et al., 2009 | Low | Unclear | Low | Low | Low | Low |
| Johnson et al., 1997 | Low | Low | Low | Low | Low | Low |
| Kim et al., 2012 | Low | Low | Low | Low | High | Low |
| Li et al., 2007 b | Low | Low | Low | Low | Low | Low |
| Li et al., 2010 a | Low | Low | Low | Low | Low | Low |
| Meng et al., 2009 | Low | Low | Low | Low | Low | Low |
| Miller et al., 1985 | Low | Low | Low | Low | Low | Low |
| Mimran et al., 1993 | Low | Unclear | Low | Low | Low | Low |
| Mohammad et al., 2009 | Low | Unclear | Low | Low | High | Low |
| Muzaale et al., 2014 | High | High | Unclear | Low | Unclear | Low |
| Najarian et al., 1992 | Low | Low | Low | Low | High | Low |
| O'Donnell et al., 1986 | Low | Unclear | Low | Low | Unclear | Low |
| Ramcharan et al., 2002 | High | Unclear | Low | Low | Unclear | High |
| Rayhan et al., 2012 | Low | Low | Low | Low | Low | Low |
| Rizvi et al., 2005 | Low | Low | Low | Low | Unclear | Low |
| Rosenblatt et al., 2008 | Low | Unclear | Low | Low | Unclear | High |
| Sahay et al., 2007 | Low | Low | Low | Low | Unclear | Low |
| Samhan et al., 1999 | Low | Unclear | Low | Low | Unclear | Low |
| Siebels et al., 2003 | Low | Low | Low | Low | Low | Low |
| Sobh et al., 1989 | Low | Low | Low | Low | Unclear | Low |
| Song et al., 2008 | Low | Low | Low | Low | Low | Low |
| Song et al., 2014 | Low | High | Low | Low | Low | Low |
| Talseth et al., 1986 | Low | Unclear | Low | Low | Low | Low |
| Tan et al., 2011 | Low | Low | Low | Low | Unclear | Low |
| Tent et al., 2010 | Low | Unclear | Low | Low | Low | Low |
| Ter Wee PM et al., 1990 | Low | Unclear | Low | Low | Unclear | Low |
| Wafa et al., 2011 | Low | Low | Low | Low | Low | Low |
| Wang et al., 2007 | Low | Unclear | Low | Low | Low | Low |
| Wang et al., 2008 | Low | Low | Low | Low | Low | Low |
| Watnick et al., 1988 | Low | Unclear | Low | Low | Low | Low |
| Wiesel et al., 1997 | Low | Low | Low | Low | Low | Low |
| Xia et al., 2002 | Low | Low | Low | Low | Low | Low |
| Xiao et al., 2012 | Low | Unclear | Low | Low | Unclear | Low |
| Yasumura et al., 1988 | Low | Unclear | Low | Low | Low | Low |
| Yazawa et al., 2011 | Low | Unclear | Low | Low | Unclear | Low |
| Zhang et al., 2009 | Low | Unclear | Low | Low | Low | Low |
| Zhao et al., 2009 | Low | Low | Low | Low | Low | Low |
| Zhao et al., 2010 | Low | Unclear | Low | High | Low | Low |
| Zhu et al., 2006 | Low | Low | Low | Low | Low | Low |

Note: Risk of bias reported for each domain using the Risk of Bias Assessment Tool for Non-randomized Studies (RoBANS).
